# EEG Theta Power Activity Reflects Workload among Army Combat Drivers: An Experimental Study

**DOI:** 10.3390/brainsci10040199

**Published:** 2020-03-28

**Authors:** Carolina Diaz-Piedra, María Victoria Sebastián, Leandro L. Di Stasi

**Affiliations:** 1 Mind, Brain, and Behavior Research Center-CIMCYC, University of Granada, Campus de Cartuja s/n, 18071 Granada; Spain; dipie@ugr.es; 2 College of Nursing & Health Innovation, Arizona State University, 550 N. 3rd St., Phoenix, AZ 85004, USA; 3 University Centre of Defence, Spanish Army Academy [Centro Universitario de la Defensa, Academia General Militar], Ctra. de Huesca, s/n, 50090 Zaragoza, Spain; msebasti@unizar.es; 4 Joint Center University of Granada - Spanish Army Training and Doctrine Command (CEMIX UGR-MADOC), C/Gran Via de Colon, 48, 18071 Granada, Spain

**Keywords:** brain activity, cognition, driving simulation, EEG, Humvee, neuroergonomics, tank

## Abstract

We aimed to evaluate the effects of mental workload variations, as a function of the road environment, on the brain activity of army drivers performing combat and non-combat scenarios in a light multirole vehicle dynamic simulator. Forty-one non-commissioned officers completed three standardized driving exercises with different terrain complexities (low, medium, and high) while we recorded their electroencephalographic (EEG) activity. We focused on variations in the theta EEG power spectrum, a well-known index of mental workload. We also assessed performance and subjective ratings of task load. The theta EEG power spectrum in the frontal, temporal, and occipital areas were higher during the most complex scenarios. Performance (number of engine stops) and subjective data supported these findings. Our findings strengthen previous results found in civilians on the relationship between driver mental workload and the theta EEG power spectrum. This suggests that EEG activity can give relevant insight into mental workload variations in an objective, unbiased fashion, even during real training and/or operations. The continuous monitoring of the warfighter not only allows instantaneous detection of over/underload but also might provide online feedback to the system (either automated equipment or the crew) to take countermeasures and prevent fatal errors.

## 1. Introduction

The armed forces represent one of the most dangerous and challenging operational systems, especially during warfare. The physiological and psychological demands of warfare are extreme and, for warfighters, are often overwhelming [1,2,3]. As operator resources are limited [4], circumstances that demand extra resources, either physical or mental, may increase the workload and, consequently, compromise operational safety [5,6]. Therefore, the armed forces are committed to finding solutions to lighten warfighters’ workload [7,8]. To reduce the physical workload, the old mechanical systems, characterized by “hand and foot” controls, have been replaced by modern, highly automatized combat platforms and advanced information systems [9,10]. Additionally, to prevent an excessive mental workload, the armed forces doctrine has introduced long and extenuating trainings, aimed at enhancing procedural and high-order cognitive skills [11,12,13,14,15]. 

Military training is of the same nature as military operations (often risky and unpredictable) and may involve extreme exertion, caloric deficit, and sleep restrictions. Overall, military training produces quantifiable and specific effects on the warfighter, including functional [16,17] and cognitive improvements [13,14,15,17,18]. Military training also has a long-lasting influence on critical everyday behaviors, such as driving [19,20]. For example, army combat drivers are taught how to safely operate different vehicles, from motorbikes to tactical vehicles, such as light multirole vehicles (LMVs), in all kinds of operational situations, from driving on sealed highways to tactical, off-road navigation. Furthermore, unlike in civilian driving, military drivers are asked and trained to multitask [18], for example, maneuvering on complex terrain environments as well as driving while monitoring communications and discriminating between enemy and friendly targets [10,21]. Thus, army combat drivers become better able to deal with overload situations [22,23]. Still, driving mishaps continue to occur among the military [24,25], with warfighters’ workload being one of the main causes [26,27] and with the LMV being one of the most accident-prone vehicles [28]. 

In the civilian domain, there has been extensive research regarding the impact of mental overload (e.g., deterioration in the psychophysiological state caused by carrying out mentally demanding tasks) on behavioral and physiological parameters among drivers [29] (for a recent review, see Ref. [30]). For instance, an increased workload level might be a consequence of (1) reduced driver resources (e.g., driving while drowsy [31]), (2) increased task demands (e.g., performing multiple tasks simultaneously [32]), or (3) reduced/unsatisfactory training [33]. Variations in driver workload are observable at the behavioral level, such as a degradation of driving performance [29], as well as at the psychophysiological level, such as an increment in electroencephalographic (EEG) theta oscillatory activity due to increased demands (distractions while driving [34]). 

### 1.1. EEG Theta Activity as an Index of Mental Workload

EEG theta oscillatory activity (4–8 Hz) appears to be functionally involved in higher brain functions including the working memory, executive control, and focused attention [35,36,37]. In particular, several laboratory studies have found a positive correlation between theta oscillatory activity and mental workload [38,39,40,41,42] (but see Ref. [43]). This would especially occur over frontal regions [44] but also on the overall scalp [42]. Classical mental workload studies have frequently used stationary tasks (e.g., [45,46]) or sophisticated EEG systems (up to 115 electrodes) to monitor EEG activity for long periods of time (i.e., large number of repetitions of a given event) (e.g., [47]). However, in situations in which a higher ecological validity is desired, it is necessary to implement easy-to-use solutions (e.g., [48]) that do not interfere with operator behavior and undermine operational safety. Pioneering works that studied ecologically-valid tasks (e.g., visual display terminal interactions [49]) and more recent studies have confirmed the sensitivity of the theta EEG power spectrum in detecting increased workload levels in operative contexts (e.g., flight simulator [2], military operations simulation facility [50]). Results obtained in driving scenarios are limited and lack consistent results, however [51]. For example, several studies have reported that the theta EEG power spectrum increased with an increased driving complexity [32,34,52], whereas a more recent study found the opposite result [53]. Overall, these results obtained from research involving non-military personnel might not be strictly applicable to the military [14,54,55]. Therefore, there is a need to better understand how mental workload affects warfighters’ psychophysiological indices as well as their driving performance [56].

### 1.2. Research Aims

Because driving is a very demanding activity involving different concurrent tasks, it is important to study the influences of diverse external factors (e.g., road geometry, traffic density) on the driver’s cognitive state and performance [57]. Thus, the aim of this study was to evaluate the effects of workload variations on the overall theta EEG power spectrum (hereafter, θ-activity) among professional army combat drivers (a young, physically fit population well trained to deal with highly demanding and stressful situations) by studying the influence of the road environment (e.g., terrain complexity), a common factor affecting driver mental workload [58]. All participants carried out realistic simulated mission scenarios with different terrain complexities that involved driving a high-fidelity dynamic LMV simulator while wearing a wearable EEG device. We also assessed driver performance (number of engine stops), as well as collected subjective ratings of the task load. Based on previous research, we hypothesized that the θ-activity and subjective ratings of the task load would increase during the most complex simulations, while performance would decrease.

## 2. Materials and Methods

### 2.1. Ethical Approval

The study followed the Code of Ethics of the World Medical Association [59]. The study was approved by the University of Granada’s Institutional Review Board (IRB approval #985/CEIH/2019) and by the Spanish Defence Medical Inspector General’s Office IRB. 

### 2.2. Participants

Forty-one professional full-time non-commissioned officers from the Spanish Army volunteered to take part in the study (ranks ranged from private to command sergeant major). All of them were professional drivers sourced from the army infantry (light and motorized) (*n* = 32), the artillery (*n* = 5), and the armored cavalry (*n* = 4) units. Inclusion criteria were (1) normal or corrected-to-normal vision and (2) a valid driving license. Exclusion criteria were (1) a medical history of significant head injury or neurological disorder and (2) low levels of arousal before the experimental session (operationalized as a score greater than 3 on the Stanford Sleepiness Scale [60], see Section 2.5). One driver was excluded for his medical history of a head injury. Another was excluded after the arousal assessment. Thus, the final sample included 39 drivers (2 females) with a mean age ± standard deviation (SD) of 31.82 ± 6.72 years. 

### 2.3. Experimental Design

The experiment followed a repeated-measures design with *terrain complexity* as the within subjects factor. Drivers underwent three driving scenarios, each of ~ 5 min long, with different terrain complexities (low, medium, and high, see Section 2.4). The θ-activity, performance (i.e., number of engine stops), and the driver’s subjective ratings of task load were the dependent variables.

### 2.4. Apparatus and Simulated Military Scenarios

#### 2.4.1. LMV “Lince” Simulator and Scenarios

**Figure 1:**
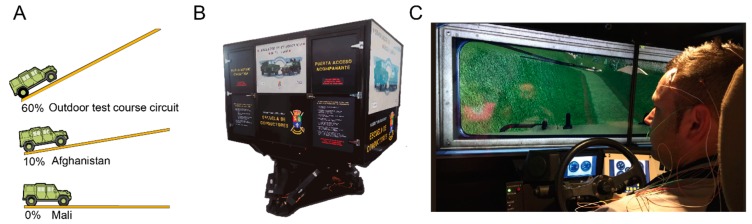
Experimental design and simulated mission scenarios. (**a**) Range of terrain complexity (maximum longitudinal slope gradient) across the three simulated scenarios: low (Mali), medium (Afghanistan), and high complexity (outdoor test course circuit) (partially adapted from www.ivecodefencevehicles.com). (**b**) The light tactical multirole vehicle (LMV) Lince simulator used in the study. An LMV Lince cabin is installed on a 6-degree motion feedback platform (partially adapted from www.simfor.es). (**c**) A participant sitting in the driver seat facing an image projection inside the LMV Lince cabin. Different electrodes are visible on his scalp.

Military simulators create complex scenarios for trainees that cannot be easily (and ethically) experienced while training in real life [56]. Here, participants drove a high-fidelity six degree-of-freedom motion-based military LMV driving simulator (Simfor-Indra S.A., Alcobendas, Spain) (henceforth, the LMV Lince simulator, see Figure 1). The LMV Lince simulator is located at the National Training Center (CENAD) “San Gregorio”, which houses several tank and LMV training simulator platforms. 

The LMV Lince simulator simulates the dynamics of the vehicle (one of the most common vehicles in international armed forces) over a vast array of terrains. The simulation environment presents participants with visual, motion, and audio cues to recreate a realistic driving experience with different levels of terrain complexity. Audio cuing includes the commander’s voice and the vehicle’s sounds (engine noise correlated to engine revolutions per minute). 

The experimental simulation included three scenarios (see Figure 1). Two of them were combat with no engagement scenarios (Mali and Afghanistan). The third one simulated a non-combat scenario (outdoor test course circuit). Because one potential major source of mental workload for drivers is scenario demands [61], we decided to manipulate this element in the simulations [62]. During one of the two combat with no engagement scenarios, drivers had to patrol a town in a Mali-based scenario. The complexity of the physical terrain was low (on-road, gravel plain circuit, with low traffic and a longitudinal slope gradient = 0%).

During the other combat with no engagement scenario, drivers went throughout a mountain port in an Afghanistan-based scenario. The complexity of the physical terrain was medium (off-road circuit with obstacle avoidance (mines, rocks, etc.) and a maximum longitudinal slope gradient of 10%). During the non-combat scenario (outdoor test course circuit), participants carried out several driving exercises. The complexity of the physical terrain was high (performing a tactic obstacle avoidance exercise and climbing a ramp with a maximum longitudinal slope gradient of 60%). We aimed to minimize our impact on normal day-to-day military duties. Therefore, we used official notional training scenarios. Even though the simulated scenarios were heterogeneous—with the outdoor test course circuit also being conceptually different—all of them were similar to the scenarios that drivers have to perform in their everyday military training.

#### 2.4.2. Electroencephalographic Recording

EEG activity was acquired with a SOMNOwatch + EEG-6 (Somnomedics GmbH, Randersacker, Germany) at a sampling rate of 256 Hz with a band pass filter of 0.1–80 Hz. For a detailed description of this wearable EEG device, see Ref. [2]. We used a monopolar montage with gold cup electrodes (Natus Neurology Incorporated—Grass Products Warwick, US) at six active scalp sites—F3, F4, T3, T4, O1, and O2—placed according to the international 10/20 system [63] and using CZ as a reference. The ground was placed at FpZ. Impedance was kept lower than 5 kΩ. We chose these specific sites keeping in mind helmet-based physiological monitoring system applications [64,65]. Electrooculography (EOG) activity was also recorded using a golden cup electrode placed in the outer canthus of the right eye (horizontal EOG channel) and another one below the left eye (vertical EOG channel) using a bipolar configuration. The device collected the raw EEG data internally. DOMINO Light software (version 14.0, Somnomedics GmbH, Randersacker, Germany) was used to export raw data to EDF+ files. From 4 out of 39 participants, due to log system failures during the EEG recordings, we only analyzed performance and subjective data.

### 2.5. Questionnaires

#### 2.5.1. Stanford Sleepiness Scale (SSS)

The SSS [60] consists of one question about alertness at a given moment. Participants had to rate their degree of alertness/sleepiness from “Feeling active, vital, alert, or wide awake” (score 1) to “No longer fighting sleep, sleep onset soon, having dream-like thoughts” (score 7), choosing one of seven statements.

#### 2.5.2. NASA-Task Load Index (NASA-TLX)

The NASA-TLX [66,67] assesses task load through six bipolar dimensions: mental demand, physical demand, temporal demand, own performance, effort, and frustration. NASA-TLX scores range between 0 and 100, with higher scores indicating a higher task load while performing the scenarios. 

### 2.6. Procedure

The experiment took place in the CENAD facility situated in Zaragoza (Spain). The participants first signed the informed consent form. Then, we collected their sociodemographic and health data. Once we had assessed the inclusion and exclusion criteria, we cleaned up the skin in the scalp and around the eyes with a slightly abrasive paste. We filled the EEG electrodes with a conductive paste and placed and secured them with collodion. When seated in the simulator, drivers filled in the SSS. After that, the participants closed their eyes for three minutes (i.e., adaption period). Then, the participants underwent a three-minute eyes-open resting state measurement. Afterwards, participants carried out the three mission scenarios. Each scenario began when the driver started the engine. After each scenario, drivers filled in the NASA-TLX. The order of appearance of the mission scenarios was counterbalanced across the drivers. After the exercises, with the participant still seated in the simulator cabin seat, he/she underwent another three-minute eyes-open resting state measurement. The overall experimental session lasted for around 30 min. All participants were familiar with the simulator but naïve to the purpose of the experiment. They were not allowed to share the contents of the experimental session with their colleagues. One private (a driving instructor not included in the experiment, the same for all the participants) played the role of the commander by giving orders and leading the communications. He also assessed performance (number of engine stops) during each mission scenario. During the experimental session, he stayed in a dedicated instructor station to start the exercises, monitor, and control the drivers’ actions (through a camera mounted inside the simulator).

### 2.7. Electroencephalographic Analyses

Raw EEG data were imported into the MatLab EEGLab software package (Mathworks Inc., Natick, MA, USA) for preprocessing and analysis. First, we filtered the data using a Butterworth filter with the −3 dB bandpass corresponding to the interval [0.5–32 Hz]. Then, we corrected for eye artifacts using a regression procedure [68] to subtract the signals recorded with the horizontal and vertical EOG channels from each data electrode. We split the continuous EEG data into periods of variable length corresponding to each of the three driving scenarios performed and then split the data for each scenario into non-overlapping two-second segments [69]. To reduce the influences of physiological and non-physiological artifacts in the analysis, we discarded data segments containing voltage values outside the [−100 µV, 100 µV] interval [2] (mean [M] of seconds discarded in each driving exercise: M _sec Mali_ = 49.28, M _sec Afghanistan_ = 45.83, M _sec circuit_ = 45.94; there were no differences among them, *p* > 0.05). We estimated the power spectra for each scenario (low, medium, and high complexity) by averaging and normalizing the Fourier transforms of the data contained in the valid segments and weighed them using a Hamming window (Bartlett’s method). Then, we obtained average values of power for the theta band (4–8 Hz) for each channel and scenario [2,5].

### 2.8. Statistical Analysis

We conducted separate analysis of variance (ANOVA) models to examine the effects of the *terrain complexity* on subjective and objective indices. To investigate whether the *terrain complexity* (three levels; low complexity (Mali), medium complexity (Afghanistan), and high complexity (outdoor test course circuit)) influenced the driving performance (i.e., number of engine stops) and subjective ratings of task load (NASA-TLX scores), we conducted two repeated-measures ANOVAs. To investigate whether the *terrain complexity* (three levels; same as before) and *channel* (six levels; F3, F4, T3, T4, O1, O2) influenced θ-activity, we conducted a repeated-measures 3 × 6 ANOVA. To test whether potential changes in θ-activity were related to the effects of fatigue due to time on task (it is likely that θ-activity increases when a person fatigues [70]), we carried out a repeated-measures 2 × 6 ANOVA with *measurement time* (eyes-open resting state measurements before and after the simulation) and *channel* (six levels; same as before) as the factors. To compare θ-activity between the EEG baseline measurement (eyes-open resting state before the simulation) and the driving exercises, we first calculated the average θ-activity for the three exercises for each channel and, then, we carried out a repeated-measures 2 × 6 ANOVA with *activity* (eyes-open resting state measurement before the simulation and driving) and *channel* (six levels; same as before) as the factors. We used the Greenhouse–Geisser correction for violations of the sphericity assumption and the Bonferroni correction for multiple comparisons. We used partial η^2^ (η_p_^2^, calculated for a repeated-measures design) to estimate the effect size. The significance level was always set at α ≤ 0.05.

## 3. Results

### 3.1. Does Terrain Complexity Have an Influence on Driving Performance and Subjective Ratings of Task Load?

Regarding performance, the repeated-measures ANOVA indicated that the effect of the *terrain complexity* on the number of engine stops was significant (*F*(1.32, 50.23) = 7.93, *p* = 0.004, η_p_^2^ = 0.17). That is, the performance was significantly worse for the Afghanistan scenario (medium complexity, mean number of engine stops (M _stops Afghanistan_) = 0.41) than for the Mali scenario (low complexity, M _stops Mali_ = 0.03, corrected *p* < 0.05). There were no significant differences in performance between the Afghanistan scenario (medium complexity, M _stops Afghanistan_ = 0.41) and the outdoor test course circuit scenario (high complexity, M _stops circuit_ = 0.15, corrected *p* > 0.05).

For the subjective ratings of task load, the repeated-measures ANOVA indicated that the effect of *terrain complexity* was significant (*F*(2,76) = 75.37, *p* < 0.001, η_p_^2^ = 0.67). After Bonferroni correction, ratings of task load were higher for the Afghanistan scenario (mean NASA-TLX score (M _NASA-TLX Afghanistan_) = 46.05) than for the outdoor test course circuit scenario (M _NASA-TLX circuit_ = 35.62) and the Mali scenario (M _NASA-TLX Mali_ = 13.31), and for the outdoor test course circuit scenario compared with the Mali scenario (all corrected *p*-values < 0.05) (see Table 1). Table 1 presents the means and standard deviations for the number of engine stops and the subjective ratings of task load for each simulated scenario.

### 3.2. Does Terrain Complexity Have an Influence on Driver θ-Activity?

The repeated-measures 3 × 6 ANOVA indicated that the main effect of the *terrain complexity* on θ-activity was significant (*F*(2,68) = 8.25, *p* = 0.001, η_p_^2^ = 0.20). The Afghanistan vs. Mali comparison (medium vs. low complexity) yielded higher levels of θ-activity for the Afghanistan scenario (mean θ-activity (M _θ-activity Afghanistan_) = 2.74 vs. M _θ-activity Mali_ = 2.49 µV^2^/Hz, corrected *p* < 0.05). The outdoor test course circuit vs. Mali comparison (high vs. low complexity) yielded higher levels of θ-activity for the outdoor test course circuit scenario (M _θ-activity circuit_ = 2.83 vs. M _θ-activity Mali_ = 2.49 µV^2^/Hz, corrected *p* < 0.05). There were no differences between the Afghanistan and outdoor test course circuit scenarios (medium vs. high complexity, *p* > 0.05). The same ANOVA also showed the expected main effect of the *channel* (*F*(1.83,62.51) = 20.72, *p* < 0.001, η_p_^2^ = 0.38). The *terrain complexity* × *channel* interaction was not significant (*p* > 0.05). 

**Figure 2:**
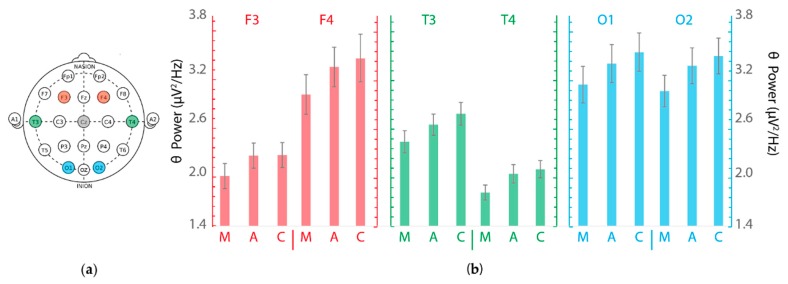
(**a**) Electroencephalographic (EEG) recording configuration. Red elements represent the frontal channels (F3 and F4), the green ones represent the temporal channels (T3 and T4), and the blue ones represent the occipital channels (O1 and O2). CZ was used as a reference. (**b**) EEG theta power (µV^2^/Hz) per channel for each simulated scenario (*n* = 35). M: Mali, low complexity; A: Afghanistan, medium complexity; C: Outdoor test course circuit, high complexity. Error bars represent the SEM across participants. There were significant main effects of *terrain complexity* and *channel* (all *p*-values < 0.05).

The repeated-measures 2 × 6 ANOVA indicated that the main effects of the *activity* and *channel* on θ-activity were significant (*F*(1, 34) = 59.38, *p* < 0.001, η_p_^2^ = 0.64 and *F*(1.67, 56.76) = 22.64, *p* < 0.001, η_p_^2^ = 0.40, respectively). θ-activity was higher for the driving exercises (average θ-activity for the three exercises) than for the eyes-open resting state measurement before the simulation in all channels. Table 2 and Figure 2 present the means and standard deviations (standard errors of the mean in the case of the figure) for θ-activity (µV^2^/Hz) per channel for each simulated scenario.

### 3.3. Does Fatigue due to Time on Task Have an Influence on θ-Activity?

As expected, we observed a significant main effect of *channel* on θ-activity (*F*(1.94, 66.13) = 16.88, *p* < 0.001, η_p_^2^ = 0.33). However, the analysis yielded non-significant differences on θ-activity for *measurement time* (*F*(1, 34) = 0.09, *p* = 0.765), which suggests that the simulated mission scenarios did not fatigue drivers (M _θ-activity pre_ = 1.85, M _θ-activity post_ = 1.87 µV^2^/Hz). The *measurement time* × *channel* interaction was not significant (*p* > 0.05). Table 2 presents the means and standard deviations for θ-activity (µV^2^/Hz) per channel for the baseline measurements (before and after the simulation).

## 4. Discussion

Army ground vehicles, as any other modern combat platform, have undergone revolutionary changes, becoming more technologically advanced [2]. Still, to work properly, these systems rely on the human factor. The operator has to deal, not only with challenging tasks requirements, but also with his/her psychophysiological state (fatigue, stress, etc.) without undermining performance. For example, driving an LMV places considerable task demands on the driver’s mental resources, not just because the dimensions of the vehicle (~7.5t × 5 m × 2 m × 2 m) make its maneuverability hard but also because of the main tasks that rely on him/her. Thus, during off-road navigation, the driver should correctly observe the terrain complexity (detection and assessment of roadway hazards) and constantly evaluate the capability of the vehicle to determine the best possible driving path [71]. These challenges are not present in on-road civilian driving. Furthermore, military drivers, unlike civilian ones, are often asked to multitask—simultaneously maintaining situational awareness, monitoring communications, and discriminating between enemy and friendly targets [10,18,21]. The information processing capacity in humans is limited and the cognitive demands imposed on warfighters are increasing. Therefore, it is important to better understand how an increased mental load affects warfighter performance. The question is not whether imposing increased cognitive demands consumes attentional resources and interferes with certain aspects of human performance, but rather how we can objectively monitor cognitive function (including brain activity) to accommodate increased cognitive demands.

### 4.1. Terrain Complexity Degrades Performance and Increases the Subjective Rating of the Task Load 

To verify the effectiveness of our experimental manipulation, we analyzed whether terrain complexity influences driver performance and subjective ratings of task load. Overall, these two indices behaved similarly: drivers experienced higher levels of task load after performing the most demanding missions (Afghanistan and the outdoor test course circuit scenarios) compared to the low complexity ones (Mali). Drivers’ performance was consistently worse (the number of errors increased) for the most complex missions. These results are in line with earlier studies using similar experimental procedures involving civilian drivers (for a recent review, see Ref. [30]). Although we categorized the Afghanistan scenario as medium complexity, it was considered to be the most demanding scenario in terms of mental workload. The inherent risk in the Afghanistan mission—including the threat of improvised explosive devices or anti-tank mines—compared to the outdoor test course circuit scenario (which was difficult but not dangerous) could have played a major role in the increased perceived task load and number of errors.

### 4.2. Terrain Complexity Increases Overall θ-Activity 

We found a higher overall θ-activity when drivers were performing the most demanding simulations (Afghanistan and the outdoor test course circuit scenarios) as compared with the low complexity one (Mali). Overall, these results strengthen previous findings on the positive correlation between the operator’s mental workload and θ-activity [2,51,72]. In particular, θ-activity in frontal areas has been associated with brain engagement in cognitive processes such as judgment, problem solving, working memory, decision making, and mathematical problem solving [73]. Here, we found that an increase in the θ-activity occurred predominantly in the right frontal electrodes (i.e., frontal θ-asymmetry). We can speculate that, since the right hemisphere is related to spatial functions (i.e., it would be dominant or specialized for the processing of visuo-spatial tasks), it would be more involved in the kind of tasks participants carry out during driving simulations [74]. Although this study was not designed to specifically investigate the topographical distribution of the θ-activity, previous studies have found hemispheric asymmetries in EEG depending on the workload demands, and it seems that such asymmetry depends on the task nature (e.g., [75]). Furthermore, temporal and occipital θ-activity behaved similarly. Very few studies have focused on those areas as potential indices of mental workload [50,76], but it seems that several cortical areas are related to different processes in the brain while dealing with complex tasks. For example, because off-road navigation performance relies heavily on the visual and motor control systems, it is plausible to explain the changes in temporal and occipital θ-activity if we consider the involvement of these areas in motor and visual processing [77,78]. 

Furthermore, another factor that might have influenced the observed θ-activity variations is the specific accidental risk level, particularly during cliff passages. The risk associated with the most demanding missions (Afghanistan and the outdoor test course circuit scenarios) might have played a relatively important role in arousal variation [79]. Thus, in our study, drivers’ levels of arousal might have adjusted according to the particular road complexity and the accident risk level of each scenario [5,80]. Furthermore, variations in arousal levels also affect θ-activity [81]. Because task complexity modulates arousal [82], the riskier scenarios might have modulated arousal levels in drivers, which, in turn, could have influenced their brain activity [2,5]. One may wonder whether the present changes in θ-activity might have resulted from increased fatigue levels due to the time spent on the task, or they might be driven by dissimilar motor—rather than complexity—demands. The first possibility seems unlikely in light of the results comparing brain activity before and after the simulated mission scenarios as well as previous research showing that the effects of driver fatigue start after around 90 min [31,83]. The second explanation would not be valid as the amount of movement-related discarded data was similar across the three scenarios.

The present study might be seen in the context of four shortcomings related to the experimental procedure, however. First, θ-activity could not discriminate between the three levels of terrain complexity. It differentiated only between the low complexity mission (Mali, longitudinal slope gradient = 0%) and the most complex missions (medium and high complexity: Afghanistan and outdoor test course circuit scenarios, maximum longitudinal slope gradient ≥10%). Although the exercises were ranked a priori by two expert military driving instructors, it is plausible that the method used to classify the three missions was not sufficiently accurate. The three missions were non-combat scenarios, but both the Afghanistan and outdoor test course circuit scenarios included the climbing of a slope. Thus, it is plausible that these two scenarios demanded similar mental resources and actions (e.g., before climbing the slope, the driver had to enable the differential-locking system) and, therefore, it was not possible to induce obvious changes in the driver’s workload levels. Future studies should disentangle this issue using scenarios/exercises that demand different mental resources and actions (e.g., dual-task paradigm). Second, the male:female ratio of our participants is not representative of the occupations within the Spanish army (about 15% female). Thus, future studies might take into account gender differences related to workload and θ-activity between army drivers [84]. Third, we did not measure motion sickness levels. Even though the drivers were observed by a camera throughout the simulation and the overall experimental session was relatively short (~30 min), discomfort feelings associated with the use of the simulator might have affected our results. Finally, in order to integrate our results with those coming from studies in the field (see Ref. [51] for a review), we implemented a classical analytical approximation method based on EEG power spectrum estimation. Recently, deep learning approaches have shown great benefits for processing EEG signals (see Ref. [85] for a recent review). Thus, future research involving the application of deep learning to EEG data might also be able to improve workload detection in dynamic and ecological situations (e.g., [86]).

## 5. Conclusions

International armies can be considered some of the largest and most diverse transportation organizations [14]. Thus, studies involving military drivers might be fundamental for improving road safety. Furthermore, international military organizations are moving toward reduced crew combat systems that will have significantly higher (cognitive) demands than current systems [87]. New wearable sensor technologies (e.g., around-the-ear electrode array [88]) enable real-time monitoring of cognitive states, which might provide objective, timely, and ecologically valid assessments of mental workload and other constructs essential to military performance [7] and road safety [57]. Thus, the investigation of the cognitive state “under fire” using advanced neuroimaging tools such as the EEG, which has excellent temporal resolution, might offer new opportunities to increase operational safety [89] in dangerous environments. Our results support the idea that EEG activity can give relevant insight into mental workload variations over time in an objective, unbiased fashion that does not interfere with performance in real-life situations [57]. The continuous monitoring of the driver’s state not only allows the instantaneous detection of over/underload but might provide online feedback to the system (either automated equipment or the crew) to take countermeasures and prevent fatal errors. Emerging neuroergonomics opportunities have great potential to improve warfighter performance and enable the development of technologies to increase their effectiveness on the battlefield [90].

## Figures and Tables

**Table 1:** Comparison of the means ± standard deviations of the subjective ratings of task load measured by the NASA-Task Load Index (NASA-TLX) and driving performance measured by the number of engine stops for the three simulated mission scenarios (*n* = 39). For the NASA-TLX, scores range from 0 to 100, with higher scores meaning a higher degree of task load. The ANOVAs yielded significant effects of the *terrain complexity* (all *p*-values < 0.05).

	Mali Scenario Low Complexity	Afghanistan Scenario Medium Complexity	Circuit Scenario High Complexity
NASA-TLX	13.31 ± 13.10	46.05 ± 19.26	35.62 ± 14.73
Engine stops	0.03 ± 0.16	0.41 ± 0.64	0.15 ± 0.37

**Table 2:** Comparison of the means ± standard deviations of the EEG theta power spectrum (θ-activity) per channel (µV2/Hz), for the baseline measurements (eyes-open resting state measurements before (pre) and after (post) the simulation) and the three simulated mission scenarios (*n* = 35). The last row shows the mean θ-activity across all six channels for each measurement/scenario.

		Eyes-Open Resting State (pre)	Mali Scenario Low Complexity	Afghanistan Scenario Medium Complexity	Circuit Scenario High Complexity	Eyes-Open Resting State (Post)
θ-activity	F_3_	1.30 ± 0.68	1.97 ± 0.84	2.20 ± 0.85	2.21 ± 0.84	1.31 ± 0.65
	F_4_	1.76 ± 1.23	2.90 ± 1.34	3.22 ± 1.33	3.32 ± 1.61	1.82 ± 1.35
	T_3_	1.68 ± 0.71	2.36 ± 0.75	2.56 ± 0.72	2.68 ± 0.77	1.71 ± 0.85
	T_4_	1.50 ± 0.57	1.78 ± 0.50	1.99 ± 0.63	2.05 ± 0.58	1.53 ± 0.60
	O_1_	2.38 ± 1.08	3.01 ± 1.23	3.26 ± 1.29	3.39 ± 1.30	2.40 ± 1.26
	O_2_	2.47 ± 1.13	2.94 ± 1.06	3.23 ± 1.21	3.34 ± 1.20	2.46 ± 1.30
Mean		1.85	2.49	2.74	2.83	1.87
